# Why eukaryotic cells use introns to enhance gene expression: Splicing reduces transcription-associated mutagenesis by inhibiting topoisomerase I cutting activity

**DOI:** 10.1186/1745-6150-6-24

**Published:** 2011-05-18

**Authors:** Deng-Ke Niu, Yu-Fei Yang

**Affiliations:** 1 Ministry of Education Key Laboratory for Biodiversity Science and Ecological Engineering, College of Life Sciences, Beijing Normal University, Beijing 100875, China

## Abstract

**Background:**

The costs and benefits of spliceosomal introns in eukaryotes have not been established. One recognized effect of intron splicing is its known enhancement of gene expression. However, the mechanism regulating such splicing-mediated expression enhancement has not been defined. Previous studies have shown that intron splicing is a time-consuming process, indicating that splicing may not reduce the time required for transcription and processing of spliced pre-mRNA molecules; rather, it might facilitate the later rounds of transcription. Because the densities of active RNA polymerase II on most genes are less than one molecule per gene, direct interactions between the splicing apparatus and transcriptional complexes (from the later rounds of transcription) are infrequent, and thus unlikely to account for splicing-mediated gene expression enhancement.

**Presentation of the hypothesis:**

The serine/arginine-rich protein SF2/ASF can inhibit the DNA topoisomerase I activity that removes negative supercoiling of DNA generated by transcription. Consequently, splicing could make genes more receptive to RNA polymerase II during the later rounds of transcription, and thus affect the frequency of gene transcription. Compared with the transcriptional enhancement mediated by strong promoters, intron-containing genes experience a lower frequency of cut-and-paste processes. The cleavage and religation activity of DNA strands by DNA topoisomerase I was recently shown to account for transcription-associated mutagenesis. Therefore, intron-mediated enhancement of gene expression could reduce transcription-associated genome instability.

**Testing the hypothesis:**

Experimentally test whether transcription-associated mutagenesis is lower in intron-containing genes than in intronless genes. Use bioinformatic analysis to check whether exons flanking lost introns have higher frequencies of short deletions.

**Implications of the hypothesis:**

The mechanism of intron-mediated enhancement proposed here may also explain the positive correlation observed between intron size and gene expression levels in unicellular organisms, and the greater number of intron containing genes in higher organisms.

**Reviewers:**

This article was reviewed by Dr Arcady Mushegian, Dr Igor B Rogozin (nominated by Dr I King Jordan) and Dr Alexey S Kondrashov. For the full reviews, please go to the Reviewer's Reports section.

## Background

### Splicing could enhance later rounds of transcription

Spliceosomal introns are a landmark feature of eukaryotic nuclear genes. However, their costs and benefits have not been fully interpreted [1-12]. One recognized effect of introns is their enhancement of gene expression. Introns and/or their splicing have been found to enhance almost every step of gene expression, from transcription to translation [13-21]. For example, intron-containing transgenes in mice are transcribed 10- to 100-fold more efficiently than the same genes lacking introns [22]. In humans and the yeast *Saccharomyces cerevisiae*, intron-containing genes produce more copies of RNA than intronless genes [18,23], and as was consistently found, removal of the introns from three essential genes in yeast significantly lowered their transcription levels [18]. Similarly, highly expressed genes were found to have higher intron densities (number of introns per kilobase of coding sequence) than weakly expressed genes in the human genome [24]. Comparison of the densities of active RNA polymerase II molecules present on genes between intron-containing genes and intronless genes in *S. cerevisiae *also showed that introns could enhance transcription (Table 1). The enhancing effects of introns on the posttranscriptional stages of gene expression are commonly attributed to proteins recruited to the mRNA during splicing [13-15,19]. By contrast, there is still no consensus on how introns and/or their splicing can increase transcription efficiency.

**Table 1:** Comparisons between the intron-containing genes and the intronless genes of *Saccharomyces cerevisiae*.

	Intron-containing genes	Intronless genes	*P *(Mann-Whitney U test)
mRNA abundance (molecules/cell)	4.9	1.3	10^-^^33^
	23 ± 38	4.2 ± 18	
Nascent transcription rate (molecules/min)	0.27	0.12	7 × 10^-28^
	0.37 ± 0.33	0.16 ± 0.20	
RNA polymerase II density (molecules/kb)	0.18	0.077	7 × 10^-28^
	0.25 ± 0.22	0.11 ± 0.13	

Data from [37]. In each item, we show the median value and the average ± standard deviation.

One possibility is that introns contain motifs that stimulate the elongation complex during transcription. It is well known that there is poor conservation of intronic sequences between most organisms. Even splicing signals such as branch sites are only loosely defined. This makes it very hard to imagine the existence of common enhancing signals in the introns present in different genes and different organisms. In the introns of *Arabidopsis thaliana*, Rose et al. [16] found loosely defined motifs that may be responsible for a gene expression enhancing effect. However, Akua et al. [25] showed that splicing is critical for the enhancing effect of the leader intron of the *Arabidopsis AtMHX *gene. Without splicing, the intron sequence displayed only low-level enhancement [25]. Recently, Morello et al. [26] constructed rice mutants that had decreased splicing efficiency, but retained the loosely defined motifs identified by Rose et al. [16]. Analysis of the mutant genes showed that the enhancement of gene expression depended heavily on the efficiency of intron splicing [26]. These observations indicate that splicing is the main contributing factor for intron-mediated enhancement of transcription.

Splicing has been shown to have extensive interactions with transcription processes and other pre-mRNA processing events [13]. So splicing may enhance gene expression by any of the aforementioned processes; from stimulating the later rounds of transcription, to facilitating the polyadenylation of the spliced pre-mRNAs.

Besides the enhancing effects of gene expression, there is also evidence indicating that splicing is the rate-limiting step in nascent mRNA production [27]. Intron splicing takes 5 to 10 min [28,29]; during this time, RNA polymerase II advances 19 to 38 kb towards the 3' end of a gene [28]. This size is far longer than almost all 3'-terminal exons in unicellular organisms. Therefore, RNA polymerase II pauses and waits for splicing to occur before finishing its transcriptional processes [30-32]. Rapidly regulated genes have been consistently found to contain few introns [33,34]. Therefore, the presence of an intron in a gene is unlikely to reduce the time required to produce an mRNA. Another possible explanation for the splicing-mediated enhancement of gene expression is that splicing mainly enhances the later rounds of transcription.

### Indirect interaction between splicing factors and later rounds of transcription

One way for splicing to enhance the later rounds of transcription is for some components of the splicing machinery to directly interact with the RNA polymerases or transcription factors operating during the later rounds of transcription [13,35]. Apparently, for such interactions and transcriptional enhancement to happen, splicing of a pre-mRNA molecule must be unfinished when the later rounds of transcription initiate. As recently established, RNA polymerases do not transcribe the 3' end of genes before finishing intron splicing [30-32]. That is, for splicing factors to interact directly in the later rounds of transcription, at least two RNA polymerase molecules should be attached to the same gene. For a ribosomal RNA gene, it is very common to have multiple RNA polymerases attached at one time and multiple transcripts synthesized simultaneously [36]. However, in protein-coding genes, it is uncommon to have multiple active RNA polymerase II molecules recruited. In *S. cerevisiae*, there are only 0.13% of genes (6 among 4,670 analyzed genes) having >2 RNA polymerase molecules/gene, and 0.86% of genes (40 among 4,670 analyzed genes) having >1 RNA polymerase molecules/gene [37]. All the 6 genes with >2 RNA polymerase molecules/gene are intronless and only 6 among the 40 genes that have >1 RNA polymerase molecules/gene contain introns. That is, only 4.5% of the 296 intron-containing genes in *S. cerevisiae *have >1 RNA polymerase molecules/gene (genome data from [38], accessed on Nov 24, 2010). Please note that the densities of polymerase II obtained by chromatin immunoprecipitation in some other studies [39] may be globally higher than the work cited here [37]. As discussed by Pelechano et al. [37], the RNA polymerase II densities reported probably included a fraction of inactive RNA polymerase II molecules, and therefore may not represent an accurate map of transcriptionally active RNA polymerase II in the yeast genome. Hence, in yeast, the enhancement of the later rounds of transcription by most of the introns is not likely to be mediated by direct interactions between splicing factors and later rounds of transcription.

Although genome-wide data on the densities of RNA polymerase II are not yet available for other species, we can roughly estimate them by steady-state mRNA abundance and mRNA stability. In yeast, the median abundance of mRNAs is 1.38 copies/cell [37]; the median half-life of mRNAs is about 20 min [40] and the median RNA polymerase II density on genes is 0.078 molecules/kb [37]. By contrast, mammalian cells have lower copy numbers of stable mRNA. In mice, the median mRNA abundance levels vary from 0.36 to 0.79 copies/cell among different cell types [41], or about 1/2.4 of the mRNA abundance in yeast. The median half-life of mouse mRNA is at least 274 min [42,43], i.e. at least 13.7 times the yeast mRNA levels. Therefore, we can estimate that the production rate of nascent transcripts in a mouse cell is 1/32.88 of that in a yeast cell. Assuming that yeasts and mammals do not differ significantly in their transcriptional elongation rates, the median RNA polymerase II density in mouse genes is 2.37 × 10^-3 ^molecules/kb. Referring to the 18.6 kb median size of mouse genes (data from Ensembl Release 60), the 2.37 × 10^-3 ^molecules/kb can be converted into 0.044 molecules/gene, a value which is lower than the 0.096 molecules/gene in yeast. From this we can estimate that genes that have recruited multiple active RNA polymerase II molecules are probably also infrequent in mice.

In both yeast and mice, therefore, direct interactions between splicing factors and the later rounds of transcription are probably infrequent events. The splicing-mediated enhancement of gene expression might be attributed to the indirect interactions occurring between splicing factors and later rounds of transcription. If future studies show that multiple active RNA polymerase II molecules on a single gene are common and direct interactions between splicing factors and later rounds of transcription are not infrequent, indirect enhancement of the later rounds of transcription by splicing would contribute less than we envision here. Nevertheless, indirect enhancement of the later rounds of transcription mediated by splicing should be explored if there is evidence for it.

In the following sections, we first propose a possible mechanism for splicing-mediated indirect enhancement of the later rounds of transcription, and then explore potential answers to the following questions: Is there any difference between the enhancement of gene expression by introns and strong promoters? What are the costs and benefits underlying the enhancement of gene expression by intron splicing?

## Presentation of the hypothesis

### Splicing makes genes less twisted and thus more accessible

It has been documented that transcription generates positive supercoiling ahead of the transcriptional assembly and negative supercoiling behind the assembly if the DNA is topologically closed [44-52]. In eukaryotes, topoisomerase I removes the negative supercoiling generated during transcription [49-55]. Extremely negatively supercoiled DNA could be observed in the transcriptionally active genes of mutants lacking topoisomerase I [45,46].

**Figure 1:**
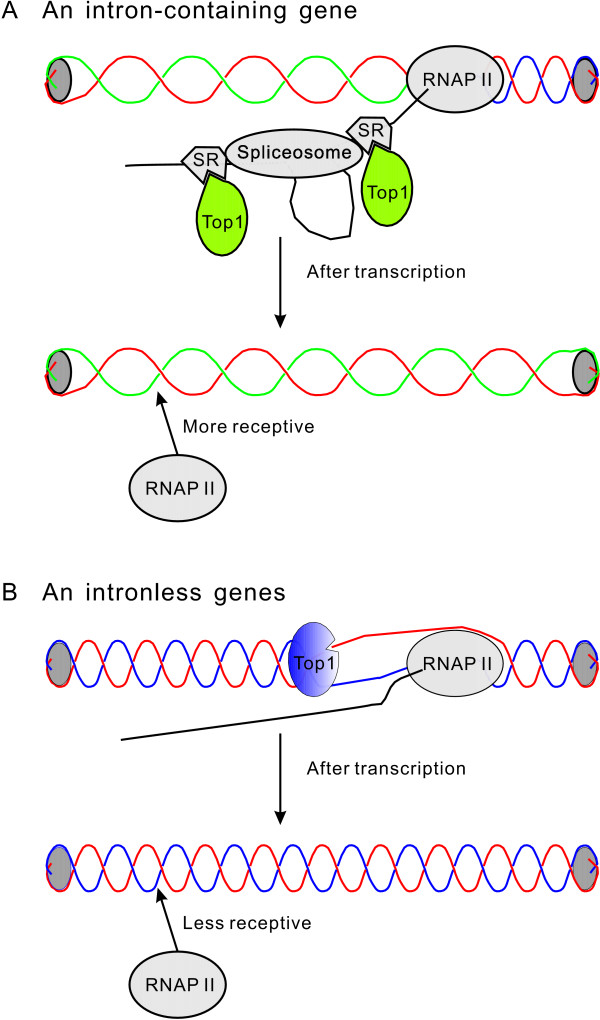
**Schematic illustration of the effect of splicing on DNA topology and accessibility to RNA polymerase II**. (A) SR proteins inhibit the cleavage and religation activity of DNA topoisomerase I (Top1). Therefore, after one round of transcription, DNA becomes less twisted and more accessible to RNA polymerase II (RNAP II). (B) In an intronless gene, Top1 actively removes the negative supercoiling generated by transcription. Transcription does not change the topological status of an intronless gene. For simplicity, nucleosomes are not shown.

In eukaryotic cells, topoisomerase I has another function; acting as a kinase to phosphorylate the serine/arginine-rich (SR) proteins like SF2/ASF [56,57]. When topoisomerase I is associated with hypophosphorylated SF2/ASF, its negative supercoiling removal activity is inhibited [58,59]. In addition, the substrate of SR protein phosphorylation, ATP, can also inhibit topoisomerase I mediated DNA cleavage [60]. Because phosphorylation of SR proteins is required for efficient splice-site recognition and the assembly of spliceosomes [61-63], we propose the following scenario; for intron splicing, SR proteins are phosphorylated by topoisomerase I, which inhibits its negative supercoiling removal activity. Because of intron splicing, the negative supercoiling generated during transcription is removed at a much lower efficiency. Consequently, intron splicing changes the transcribed gene into a less twisted state (Figure 1A). By contrast, in intronless genes, the negative supercoiling generated by transcription is removed efficiently by topoisomerase I, and so the gene reverts back to its original topological status after transcription (Figure 1B). The binding of proteins to less twisted DNA is thermodynamically favored, and thus the separation of two strands is facilitated [49,50,64,65].

In summary, we propose that intron splicing inhibits the topoisomerase I negative supercoiling removal activity, which consequently facilitates later rounds of transcription. Consistent with this hypothesis, the intron-containing genes of *S. cerevisiae *have more active RNA polymerase II molecules attached to them and higher nascent transcription rates than intronless genes (Table 1).

### Both splicing and strong promoters could enhance transcription

In rice, it has been shown that the presence of an efficiently spliced intron could compensate for the reduced transcription level resulting from a weak promoter [26]. Among the 124 cytoplasmic ribosomal protein genes in *S. cerevisiae*, 94 are intron-containing and 30 are intronless (data from [38], accessed on Nov 24, 2010). Analysis of their mRNA abundance levels did not reveal any significant differences between intron-containing ribosomal protein genes and intronless ribosomal protein genes [66]. Apparently, intronless ribosomal protein genes have their own strategies to enhance their transcription levels. The most likely strategy in this case is to have stronger promoters. Hence, eukaryotic cells could elevate their gene transcription levels by having introns or by having strong promoters.

### Benefits of splicing: avoiding the dark side of topoisomerase I

If eukaryotic cells could elevate their transcription levels simply by having strong promoters, why do they bother to use splicing, which is a complex and energy expensive process [67]? The most likely answer is that splicing must be beneficial to eukaryotic cells. Although many benefits of introns and intron splicing have been suggested [5-10], here we propose a new one based on the scenario proposed in previous sections of this paper.

To remove negative supercoiling in DNA, topoisomerase I has to generate breaks in one of its strands. This process poses a potential threat to genome integrity. In many unfavorable conditions, this threat is magnified to cause genome instability [68]. Nitiss et al. [69] over-expressed yeast topoisomerase I and found that the genome became hypersensitive to methyl methanesulfonate and other DNA-damaging agents. For many years, high transcriptional rates have been found to be associated with genetic instability [70,71]. Recently, two groups consistently found that deletion of topoisomerase I could completely eliminate transcription-associated short DNA deletions [72,73].

If splicing could inhibit topoisomerase I DNA cleavage and religation activity, eukaryotic cells could avoid the dark side of topoisomerase I, while maintaining a high level of transcriptional activity. However, cells lacking introns and splicing activity do not necessarily exhibit obvious growth rate defects. Indeed, the deletion of most introns has been found to have no significant effects on cell growth, and, in the laboratory setting at least, introns appear to be nonessential [74]. If our hypothesis is correct, intron deletion would increase the risk of genome instability, making it an unlikely evolutionary favored strategy.

## Testing the hypothesis

If our hypothesis is correct, splicing inhibits topoisomerase I DNA cleavage activity, thus reducing the frequency of transcription-associated short DNA deletions. That is, transcription-associated mutagenesis would be much lower in intron-containing than intronless genes. Experimental approaches that block intron recognition and splicing would strengthen the validity of transcription-associated mutagenesis.

Because topoisomerase-I-associated damage causes mainly short DNA deletions [72,73], this would result in the exons flanking lost introns becoming shorter over evolutionary time. Bioinformatic analysis of the frequency of short DNA deletions in the exons flanking lost introns may provide evidence for this hypothesis.

## Implications of the hypothesis

### Beneficial since early eukaryotes

In lower organisms with small introns, it was believed that introns contained all the information required for accurate splicing, a mechanism called intron definition [75]. By contrast, exon sequences play major roles in the recognition of intron/exon structures in organisms with long introns (termed exon definition). In *Drosophila melanogaster*, both short and long introns are typically found. Fox-Walsh et al. [76] demonstrated that intron definition becomes less efficient as intron size increases. The threshold for cessation of recognition across introns is 200 to 250 nt. Indeed, in some unicellular organisms, long introns are not unusual. For example, there are 143 introns >200 nt and 139 introns >250 nt in *S. cerevisiae *(data from [38], accessed on Nov 24, 2010) and 290 introns >200 nt and 173 introns >250 nt in *Schizosaccharomyces pombe *(data from [77], accessed on Jan 11, 2011). In *S. pombe*, an SR protein named Srp2p was reported to attach to exonic sequences and promote recognition and splicing of introns that had weak intronic splicing signals [78]. In *S. cerevisiae*, an SR-like protein called Npl3 is required for efficient splicing of many pre-mRNAs [79]. In addition, there is evidence that SR proteins also participate in intron definition in human cells [80]. Therefore, SR and SR-like proteins are likely to exist in most, if not all eukaryotes, with the purpose of facilitating the splicing of weak introns [62,81,82]. If weak splicing signals and SR and SR-like splicing facilitators are ancestral [83], we could argue that the splicing-mediated enhancement of gene transcription might have been beneficial, since the early evolution of eukaryotic cells.

There is still no evidence, however, that the SR-like protein Npl3 inhibits the negative supercoiling removal activity of topoisomerase I in *S. cerevisiae*. This is a gap in our hypothesis.

### Long introns: weak in splicing thus efficient in transcriptional enhancement

Another insight from the results of Fox-Walsh et al. [76] is that long introns are weak, and thus require more help from exonic splicing signals. Long intron splicing is more likely to require the recruitment SR or SR-like proteins. According to our hypothesis, long introns should be more efficient in transcriptional enhancement than short introns. Hence we would expect there to be a positive correlation between intron size and gene expression levels. This is, in fact, what has been widely observed in unicellular organisms [18,33,84,85]. Early studies also supported the same trend in plants [33,86,87]. However, a later study in plants *A. thaliana *and *Oryza sativa *showed that genes with longer introns were weakly expressed [88], a trend that is consistently observed in animals [2,87,89,90]. A recent more detailed analysis of four multicellular organisms (*Homo sapiens*, *Caenorhabditis elegans*, *D. melanogaster *and *A. thaliana*) revealed an approximate bell-shaped relationship between intron size and gene expression levels [91]. With increasing expression levels, introns first become longer, but eventually become shorter [91]. Besides the SR protein SF2/ASF, some other splicing-related proteins are also found to interact with human topoisomerase I [92]. For example, PSF/p54^nrb ^activates topoisomerase I to remove negative supercoiling [93]. The splicing apparatuses of multicellular organisms are more complex than those of unicellular organisms like yeast [67]. It is therefore reasonable to assume that unknown interactions between splicing and transcription exist in higher organisms. The evolution of intron size in higher organisms is unlikely to be neatly explained by a single factor such as that proposed in this paper.

### The cost of introns

If introns only confer the benefits proposed by ourselves and others [5-10], the loss of introns would be selected against during evolution. In cases where the cost of an intron exceeds its benefit(s), loss of the intron would be positively selected for. And if the cost(s) only just balances the benefit(s), intron loss may be fixed in evolution by random drift. Many cases of intron losse have been documented in evolution [94-105]. So, if our hypothesis is correct, introns and/or their splicing should also confer a considerable cost to an organism. It has been shown that intron splicing is a time-consuming process [27-29], and so introns are selected against in rapidly regulated genes [3,33]. Crucially, transcription and intron splicing consume energy. Thus, in organisms with very large populations, like *S. cerevisiae*, the energetic cost of a long intron in a highly expressed gene is a burden visible to natural selection [106].

## Competing interests

The authors declare that they have no competing interests.

## Authors' contributions

DKN conceived the hypothesis and wrote the original draft; YFY collected the genome and expression data and modified the manuscript; both authors read and approved the final text.

## Reviewers' comments

### Reviewer 1

Dr Arcady Mushegian, Stowers Institute of Medical Research, USA

The origin of eukaryotic introns is most likely explained from the mechanistic point of view by group II intron invasion from the mitochondrial ancestor, and from population point of view by weak purifying selection in populations with small *Ne*. What promotes intron persistence in all eukaryotes, aside from small *Ne*, is an open question. The authors argue that a factor here is the ability of one of the SR proteins to inhibit topo I activity, thus reducing mutation rate. This is an interesting hypothesis compatible with some of the observed data on correlation between intron length, expression strength, polymerase occupancy, etc. I request, however, that the others state more explicitly their opinion on when in the course of evolution this inhibition arose - do I understand it correctly that it had to be an ancient property, and if so, has this been borne out by pinpointing the origin of the SR factor in question, or by showing that this is a general property of many SR factors, not the serendipitous advantage of this particular one?

**Authors' response**: *This is a very important question raised about our hypothesis. We would also like to be able to see the potential benefit(s) that might have driven the origin and evolution of spliceosomal introns. Unfortunately, we are unable to speculate further about this at this time, because very little is known about the origin and early evolution of spliceosomal introns and SR proteins. Further evidence is required to offer a more explicit opinion*.

### Reviewer 2

Dr Igor B Rogozin, NCBI/NLM/NIH, USA (nominated by Dr I King Jordan)

The paper discusses various issues related to the positive correlation between intron size and gene expression which is observed in unicellular organisms and some multicellular organisms. This correlation is not particularly strong and have various explanations, for example, longer introns may be more efficiently spliced out or may be splicing is important for an efficient transport of mRNA. The authors suggested their own hypothesis:

"If splicing could inhibit topoisomerase I DNA cleavage and religation activity, eukaryotic cells could avoid the dark side of topoisomerase I, while maintaining a high level of transcriptional activity. However, cells lacking introns and splicing activity do not necessarily exhibit obvious growth rate defects. Indeed, the deletion of most introns has been found to have no significant effects on cell growth, and, in the laboratory setting at least, introns appear to be nonessential [74]. If our hypothesis is correct, intron deletion would increase the risk of genome instability, making it an unlikely evolutionary favored strategy."

I think that by the "the genetic risk" the authors mean the increased rate of spontaneous mutations. In general, I do not think that the transcription-associated mutagenesis is different from other sources of spontaneous mutations. I do not see any connection between the transcription-coupled mutagenesis/repair and introns. Some unicellular eukaryotes have a few introns, prokaryotes without introns (self-splicing introns) are doing just fine. I do not think that they are under any "genetic risk". Thus the transcription-coupled mutagenesis/repair is unlikely to be an important factor in evolution of the exon/intron structure.

**Authors' response**: *We consider that our hypothesis may provide some insight about the correlation between intron size and gene expression levels. The main question we want to address is the correlation observed between the presence of an intron and the effect it might have on the gene expression level; such a correlation has been found in many genome-wide bioinformatic analyses and transgenic analyses *[18,22-24,107-111].

*We also do not know if any connection between transcription-coupled mutagenesis/repair and introns exists. But, in the light of such a hypothesis, we would seek to investigate this further*.

"Some unicellular eukaryotes have a few introns, prokaryotes without introns (self-splicing introns) are doing just fine." *There are two possible explanations here. The first is the widely held opinion that introns are slightly deleterious, and so the presence/absence of introns depends mainly on the efficiency of natural selection. The second is that introns are abundant in some organisms (like humans) and some genes from intron-rare organisms (like the ribosomal proteins genes of *S. cerevisiae*) because of the distinctiveness of these organisms and these genes. These organisms may be less able to tolerate genetic risk than others. And these genes (e.g., ribosomal protein coding genes and other evolutionarily conserved genes) may be less tolerant of genetic risks than other genes. In *S. cerevisiae, *only 3.1% of the nuclear genes contain introns, but the majority (75.8%) of cytoplasmic ribosomal protein genes have introns (genome data from *[38], *accessed on Nov 24, 2010). In addition, Dr. Rogozin and colleagues have reported that evolutionarily conserved genes tend to have more introns *[112]. *Certainly, these observations are consistent with our hypothesis, but not proof per se. However, there is, to the best of our knowledge, no convincing evidence to reject the hypothesis that introns are retained in some organisms and some genes because of their intolerance of genetic risk*.

The authors suggested two ways to test the hypothesis:

"If our hypothesis is correct, splicing inhibits topoisomerase I DNA cleavage activity, thus reducing the frequency of transcription-associated short DNA deletions. That is, transcription-associated mutagenesis would be much lower in intron-containing than intronless genes. Experimental approaches that block intron recognition and splicing would strengthen the validity of transcription-associated mutagenesis.

Because topoisomerase-I-associated damage causes mainly short DNA deletions [72,73], this would result in the exons flanking lost introns becoming shorter over evolutionary time. Bioinformatic analysis of the frequency of short DNA deletions in the exons flanking lost introns may provide evidence for this hypothesis."

However, the authors did not try to find any support for the hypothesis. I think that if the authors did not do the suggested analyses by themselves, nobody is going to do it.

**Authors' response**: *We thank Dr. Rogozin for reminding us of this. We did not want to write a research article with a very long introduction, but preferred to formulate a hypothesis and then (after some studies) write a concise research article on the subject*.

I suggest to readers of this paper to consider it as a review paper rather than a hypothesis paper.

**Authors' response**: *We do not completely disagree with this suggestion. In fact, we ourselves have often derived more benefit from reading the background and introduction sections than the hypothesis section of some hypothesis papers*.

### Reviewer 3

Dr Alexey S Kondrashov, Department of Ecology and Evolutionary Biology, The University of Michigan, USA

This reviewer provided no comments for publication.
